# *Padina boryana* mediated green synthesis of crystalline palladium nanoparticles as potential nanodrug against multidrug resistant bacteria and cancer cells

**DOI:** 10.1038/s41598-021-84794-6

**Published:** 2021-03-08

**Authors:** Hana Sonbol, Fuad Ameen, Sami AlYahya, Abobakr Almansob, Suaad Alwakeel

**Affiliations:** 1 Department of Biology, College of Science, Princess Nourah Bint Abdulrahman University, Riyadh, Saudi Arabia; 2 Department of Botany & Microbiology, College of Science, King Saud University, Riyadh, 11451 Saudi Arabia; 3 National Center for Biotechnology, King Abdulaziz City for Science & Technology, Riyadh, Saudi Arabia

**Keywords:** Biological techniques, Nanoscience and technology

## Abstract

Green synthesized nanoparticles (NPs) have emerged as a new and promising alternative to overcome the drug resistance problem. Peculiar nano-specific features of palladium NPs (Pd-NPs) offer invaluable possibilities for clinical treatment. Due to the development of multi-drug resistance (MDR) in pathogenic bacteria and the prevalence of cancers, use of algae-mediated Pd-NPs could be a prospective substitute. Therefore, Pd-NPs were synthesized by a one-step, cost-effective, and environmentally friendly green method using the extract from a brown alga, *Padina boryana* (PB-extract), and evaluated for their antibacterial, antibiofilm, and anticancer activities. Pd-NPs were physicochemically characterized for size, shape, morphology, surface area, charge, atomic composition, crystal structure, and capping of Pd-NPs by PB-extract biomolecules by various techniques. The data revealed crystalline Pd-NPs with an average diameter of 8.7 nm, crystal size/structure of 11.16 nm/face-centered cubic, lattice *d*-spacing of 0.226 nm, 28.31% as atomic percentage, surface area of 16.1 m^2^/g, hydrodynamic size of 48 nm, and zeta-potential of − 28.7 ± 1.6 mV. Fourier-transform infrared spectroscopy (FT-IR) analysis revealed the role of PB-extract in capping of Pd-NPs by various functional groups such as –OH, C=C, C–O, and C–N from phenols, aliphatic hydrocarbons, aromatic rings, and aliphatic amine. Out of 31, 23 compounds were found involved in biosynthesis by Gas chromatography–mass spectrometry (GC–MS) analysis. Isolated strains were identified as MDR *Staphylococcus aureus*, *Escherichia fergusonii*, *Acinetobacter pittii, Pseudomonas aeruginosa, Aeromonas enteropelogenes*, and *Proteus mirabilis* and Pd-NPs exhibited strong antibacterial/antibiofilm activities against them with minimum inhibitory concentration (MIC) in the range of 62.5–125 μg/mL. Moreover, cell viability assays showed concentration-dependent anti-proliferation of breast cancer MCF-7 cells. Pd-NPs also enhanced mRNA expression of apoptotic marker genes in the order: *p53* (5.5-folds) > *bax* (3.5-folds) > *caspase-3* (3-folds) > *caspase-9* (2-folds) at 125 μg/mL. This study suggested the possible role of PB-extract capped Pd-NPs for successful clinical management of MDR pathogens and breast cancer cells.

## Introduction

Nanotechnology manipulating the matter at molecular scale has tremendously revolutionized the field of science^1^. Nanoparticles (NPs) with dimeter between 1–100 nm possess distinguished physicochemical properties over bulk-materials including higher surface area to volume (S/V) ratio, surface energy, and chemical reactivity^2^. Physical or chemical methods of NPs synthesis use harmful toxic substances and high temperatures/pressure which make them environmentally unfriendly and expensive^3^. Moreover, NPs synthesized using toxic chemicals when used medically can also damage human health. Therefore, water extracts prepared from various organisms such as plants (fruit, leaves, roots, and bark)^4^, algae (cyanobacteria and higher algae)^5^, bacteria (cell supernatant)^6^, and fungi (extra mycelial/cellular biomolecules)^7^ have been utilized to fabricate different metal-based NPs. Among these, algae mediated synthesis of NPs is relatively less explored. This approach is not only economical but also poses negligible risk to environmental health^8^. Besides, algae can be found easily in bulk quantities at coastal sites such as coastal regions of Saudi Arabia^9,10^. Therefore, metal based NPs synthesis using algal extracts have attracted greater attention for various applications. For example, Ag-NPs from *Botryococcus braunii*^11^, Au-NPs from *Egregia sp*^12^, CuO-NPs from *Bifurcaria bifurcata*^13^, and Fe_3_O_4_-NPs from *Sargassum acinarium*^14^ have been synthesized. In particular, brown algal (seaweed) species have scarcely been reported mechanistically to produce NPs of platinum group. Few studies done in recent past have documented the synthesis of Pt-NPs from brown seaweed *Padina gymnospora*^15^ and Pd-NPs from blue-green algae *Spirulina platensis*^16^. So far no study has reported the mechanism of synthesis of Pd-NPs from brown alga *Padina boryana* and their antibacterial, antibiofilm, and anticancer potential.

Multidrug resistance (MDR) in clinical pathogenic bacteria and cancerous cells is of huge concern due to growing worldwide incidences of MDR and low efficacy of available drugs^17,18^. Among the clinical bacterial pathogens, *S. aureus*, *P. aeruginosa*, *E. fergusonii*, *A. pittii*, *A. enteropelogenes*, and *P. mirabilis* have affected the human population due to the development of antibiotic resistance mechanisms in bacteria^19^. Also, breast cancer is a recognized cause of death worldwide and has been the fourth in the list of cancer triggered mortalities in the USA alone^20^. Therefore, new nano-based strategies such as algal-derived metabolites capped NPs could be adopted after successful clinical trials.

The Pd-NPs have found applications in catalysis for wastewater remediation^21^, degradation of pollutant dyes^22^, bactericidal and antifungal activities, and anticancer activities^23^. Indeed, there are other metal and metal oxide NPs being used in various biomedical applications including antibacterial and anticancer. These NPs are Au, Ag, Fe, ZnO, and CuO etc. However, the noble Pd-NPs have shown excellent physicochemical features like high photocatalytic activity, remarkable chemical stability, good thermal stability, optical and electronic properties^24^. Especially in pharmaceutical industry, Pd-NPs catalyse many different reactions including catalysis resulting in C–C bond formation and oxidation processes^25,26^. As compared to other nanoscale materials such as Au and Ag nanospheres, plasmonic Pd nanospheres have shown higher susceptibility to refractive index changes^27^. Besides, Pd-NPs exert prominent inhibition of several cancer cell lines and bacterial strains. Besides with multifarious applications, Pd-NPs are able to enhance the biomedical diagnosis and clinical therapies with minimum side effects. However, due to thermodynamic instability, Pd-NPs usually form aggregates which limit their broader biological applications. To this end, algal extract mediated fabrication might stabilize the Pd-NPs by efficient surface adsorption of algal biomolecules. Bioactive molecules present in algal extracts such as polysaccharides, proteins, fatty acids, phenolics, flavonoids, etc.^28^ can efficiently reduce Pd^2+^ ions and functionalize/cap growing seeds of NPs thus reducing the size of final Pd-NPs. Extracts of *P. boryana* are also rich in biologically active compounds including compounds with high phenolic content^29^ including flavonoids and tannins, proteins and steroids^30^ with antimicrobial activities. *P. boryana* extract also inhibits cellular tyrosinase levels and melanin synthesis which suggests its cosmeceutical and medicinal applications^31^.

Considering the clinical importance of *P. boryana* extract and Pd-NPs, this study for the first time was designed comprehensively to achieve the following objectives: (i) synthesis and capping of Pd-NPs by biomolecules of *P. boryana* extract and its characterization by UV–visible, X-ray diffraction (XRD), and energy dispersive X-ray (EDX) spectroscopy, scanning and transmission electron microscopy (SEM/TEM), dynamic light scattering (DLS), and zeta-potential, (ii) determination of *P. boryana* biomolecules adsorption on the surface of Pd-NPs and their role in capping by FT-IR spectroscopic and GC–MS analyses, (iii) isolation and identification of multi drug-resistant clinical bacteria, (iv) assessment of bacterial growth and biofilm inhibiting potential of PB-capped Pd-NPs, and (v) MCF-7 cell anti-proliferative and apoptosis inducing potential of PB-capped Pd-NPs.

## Materials and methods

### Collection and preparation of aqueous extract of *P. boryana*

Brown seaweed of *P. boryana* was collected from the Saudi coastal region of the Arabian Gulf (latitude 26° 11ʹ 45.8ʺ N and longitude 50° 01ʹ 37.8ʺ E). Samples were transported to the laboratory in sterile polystyrene containers. Collected samples were washed well several times with running tap water followed by sterile deionized water (DIW) to remove unwanted debris. Samples were shade dried for 40 days in an incubator at 28 °C. Dried samples were powdered using a mortar and pestle and sieved through a mesh to obtain a fine powder. To prepare the extract, dried *P. boryana* powder (5 g) was added to sterile 100 mL DIW followed by ultrasonication at low amplitude (20%) for 40 min. The mixture was then incubated on a magnetic stirrer at 120 r/min for 24 h. The extract was filtered first through a sterile blotting paper and then sterile Whatman No. 1 filter discs using a vacuum filtration assembly. Aqueous extract of *P. boryana* (PB extract) was stored in 50 mL aliquots at − 20 °C until further use.

### Green fabrication of PB-capped Pd-NPs

Pd-NPs were prepared by facile one-pot fabrication method using green PB-extract. A 5 mL of PB-extract was mixed with 100 mL of 10 mM disodium tetrachloropalladate (II) (Na_2_PdCl_4_) prepared in sterile DIW procured from Sigma Aldrich, USA (product code-379808 with 99.99% purity). The mixture was vigorously stirred using a magnetic stirrer at 200 r/min for 2 h maintaining the stirring temperature at 60 °C. Sterile experimental conditions were made throughout the synthesis and, to avoid the photo mediated changes in PB-extract, dark condition was maintained during Pd-NPs synthesis by wrapping the glass beakers by 0.024 mm thick aluminum foil. The color of PB-extract was pale yellow which became dark brown denoting the successful synthesis of Pd-NPs. After completion of the reaction, the dark solution of Pd-NPs was centrifuged at 10,000 r/min for 30 min and the pellet was separated from the supernatant. Pellets were collected and supernatants were again centrifuged at 12,000 r/min for 30 min to collect remaining smaller sized Pd-NPs. The pellets were washed at least thrice with sterile DIW followed by freeze-drying. Freeze-dried powder of PB-capped Pd-NPs was stored in cleaned brown bottles till characterization and assessment of biocidal activities.

### Physicochemical characterization of PB extract-capped Pd-NPs

#### UV–Vis, EDX, and SEM analyses

Liquid samples of PB-extract, Na_2_PdCl_4_, and PB extract-capped Pd-NPs were checked for their absorption in the UV–visible range (280–600 nm) using double beam operation of PerkinElmer Lambda 35 spectrophotometer (Waltman, MA, USA). To measure the surface morphology, the powder of Pd-NPs was put on to a carbon tape and aluminum stub carrying carbon tape was analyzed by a scanning electron microscope attached with EDX at an accelerating voltage of 15 kV (SEM–EDS; JEOL-64000, Tokyo, Japan).

#### Structure, shape, and size determination of PB extract-capped Pd-NPs

Transmission electron microscope (JEM-1011, JEOL, Tokyo, Japan) was used for the determination of average diameter and shape of Pd-NPs at 200 kV energy. The aqueous suspension (15 μL) of Pd-NPs was put on a Cu-grid followed by drying at 80 °C for 5 h. Prepared grids were analyzed by TEM. To check the crystallinity and phase purity, XRD analysis was performed on Bruker D8 Discover instrument. Cu-Kα radiation (λ = 1.54 Å) was used to obtain the diffraction pattern and data was recorded at 20–80° two-theta (2θ) angle.

#### Hydrodynamic size, zeta-potential, and surface area measurement

To obtain the hydrodynamic size, 50 µg/mL suspension of Pd-NPs was prepared in DIW and ultrasonicated at 40% amplitude for 15 min. The Pd-NPs suspension was then subjected to analysis by a Zeta Sizer Nano-ZS90, Malvern, UK. The zeta-potential of Pd-NPs was recorded as an average of 20 readings. Specific surface area measurement of PB-capped Pd-NPs was done following Brunauer–Emmett–Teller (BET) analysis using Autosorb-iQ-MP/XR surface area analyzer (Quantachrome Instruments, USA).

#### Determination of surface functional groups and compounds of PB-extract and Pd-NPs

To detect the adsorption of functional groups, FT-IR analysis of PB-extract and Pd-NPs was recorded in attenuated total reflectance (ATR) mode on the PerkinElmer system 2000 instrument. The spectra for each sample were scanned three times and average values of percent transmittance were plotted against wavelength (4000–400 cm^−1^). GC–MS analysis of hexane extracts of *P. boryana* and PB-extract capped Pd-NPs was performed on Shimadzu QP-2010 Plus with Thermal Desorption System TD-20 instrument. Conditions for analysis were kept as follows: He flow at 1.2 mL/min, oven temperature from 80 to 260 °C at 4 °C/min, for 5 min, interface/inlet temperature were set as 280/250 °C. A 0.2 mL solution was injected at a 10:1 split ration at 70 eV. Data for signals obtained for various molecules such as retention time, peak percentage area, molecular mass, etc. was recorded based on the interpretation of the National Institute of Standards and Technology (NIST) library.

### Isolation and characterization of clinical bacterial pathogens

Samples for isolation of clinical bacteria were collected from fluid and sputum of immunocompromised patients diagnosed with respiratory infections following our earlier described method^32^. After biochemical and morphological identification, antibiotic-resistant isolated cultures were molecularly characterized by partially sequencing 16S rRNA gene using universal primers 785F (5ʹ -GGATTAGATACCCTGGTA-3ʹ) and 907R (5ʹ-CCGTCAATTCMTTTRAGTTT-3ʹ) following our previously demonstrated methods of 16S rDNA amplification and Sanger’s dideoxy sequencing. The sequences were processed using BioEdit software 7.2.4. For similarity search, the BLASTn search tool of NCBI was used. The processed sequences for isolated bacterial strains were submitted to the GenBank database and accession numbers were obtained. The strains were stored in Luria Bertani (LB) broth supplemented with glycerol (40%) at − 70 °C in duplicates until further use. Detailed method of phylogenetic analysis can be found in supplementary information.

### Antimicrobial drug resistance profiling of bacterial isolates by disc susceptibility test

Drug resistance was checked by Kirby-Bauer’s disc diffusion assay of antibiotics with known disc potency on LB agar media following the guidelines of the Clinical and Laboratory Standards Institute (CLSI, 2016)^33^. The size of the inhibition zone around antibiotic discs was measured and captioned as sensitive (S), intermediate (I), and resistant (R) based on the manufacturer’s criteria. Control strains of *E. coli* ATCC-25922 and *P. aeruginosa* ATCC-27853 were used.

### Evaluation of the antibacterial potential of PB-capped Pd-NPs

#### Antibacterial well-diffusion assay

The PB-capped Pd-NPs were screened for their antibacterial potential by agar well diffusion method against *S. aureus* strain FA-1, *E. fergusonii* strain FA-5, *A. pittii* strain FA-6, *P. aeruginosa* strain FA-7, *A. enteropelogenes* strain FA-8, and *P. mirabilis* strain FA-9. A 100 μL culture (~ 1 × 10^8^ cells/mL) of each bacterium was separately spread plated on LB agar plates. Wells of 8 mm size were cut and the base was sealed with 0.6% agar. A 100 μL from Pd-NPs stock solution (1 mg/mL) was added to wells. Negative (100 μL PB-extract) and positive (gentamicin 10 µg/disc) controls were also incorporated. Petri plates were incubated for 24 h at 37 °C and results of inhibition zones were compared.

#### Determination of MIC by colony forming unit (CFU) count method

A 20 mL of LB broth in 50 mL capacity conical flasks amended with 7.81–250 μg PB-extract capped Pd-NPs/mL) was inoculated with young bacterial cultures in triplicates. Flasks were incubated in a shaker incubator at 120 r/min constant stirring, 37 °C for 24 h. After 24 h incubation, 100 μL culture from each test concentration for each test bacterium was spread plated on LB agar and after incubation under static conditions, the number of colonies was counted and converted to CFU/mL. The concentration at which the number of cells was negligible, was taken as MIC.

#### Bacterial growth measurement as a function of PB-capped Pd-NPs concentration

To observe the concentration-dependent growth inhibition of bacterial cultures, the cultures were grown with 7.81–250 µgPd-NPs/mL in LB broth as mentioned for MIC determination. Log_10_ CFU/mL were graphically plotted with increasing concentration of PB-capped Pd-NPs.

#### Impact of PB-capped Pd-NPs on the bacterial cell membrane

A fluorescence-based method was used to detect membrane compromised cells. Bacterial cells were grown in LB broth for 12 h. Cultures were centrifuged, cell pellets were washed with 1X sterile phosphate buffered saline (PBS), and re-suspended in 5 ml PBS followed by treatment with Pd-NPs at 7.81–250 μg/mL at continuous stirring (120 r/min) for 2 h. Thereafter, a fluorescent DNA tag called propidium iodide (PI) was added maintaining the final concentration of PI as 25 μM. After 20 min of incubation at room temperature, cells were again washed thrice with sterile 1X PBS to remove the unbound PI, and slides were prepared for confocal laser scanning microscopy (CLSM) and visualized under Leica TCS SPE microscope. The number of cells with membrane defects emitting red fluorescence of PI were enumerated from the three best preparations and mean values were plotted as a function of Pd-NPs concentration.

### Impact of PB-capped Pd-NPs on biofilm-forming ability of bacterial isolates

Biofilm-formation was determined by crystal violet (CV) microtiter plate method. Bacterial isolates were first checked for biofilm formation and compared with biofilms of standard biofilm-forming strains of *E. coli* ATCC-25922, *S. aureus* ATCC-9144, and *P. aeruginosa* ATCC-27853 by broth dilution method and optical microscopy. To determine the absorbance based biofilm formation, 0.1 mL young culture (~ 1 × 10^7^ CFU/mL) grown in LB broth from each isolate was added to respective microtiter wells in triplicates. The cultures were added with appropriate stock concentration of PB-extract capped Pd-NPs maintaining the final concentration as 3.9–250 μg/mL. Two negative controls: (i) broth only, (ii) broth + 3.9–250 μg/mL Pd-NPs, and one positive control (bacterial culture only) were run in parallel. A 2% sucrose was also added to induce the production of exopolysaccharides for biofilm formation. After incubation for 48 h at 37 °C, the wells were rinsed gently sterile PBS and then added with 1% CV solution (200 μL) followed by incubation for 20 min. Wells were again rinsed thrice with PBS, dried under laminar airflow, and the CV remained in biofilm was solubilized by the addition of 95% ethanol. The absorbance at 600 nm was measured. Bare surface Pd-NPs (bare-PdNPs) procured from Sigma-Aldrich, USA (product code-686,468) with an average particle diameter of < 25 nm (determined by TEM) were also tested at 15.62, 31.25, 62.5 μg/mL and the results were compared with PB-extract capped Pd-NPs. The data was interpreted as percent biofilm formation over positive control.

### Cellular anti-cancer activity and apoptosis induced by PB-capped Pd-NPs in MCF-7 cells

The anti-cancer potential of PB-capped Pd-NPs was determined in vitro by 3-(4,5-dimethylthiazol-2-yl)-2,5-diphenyl tetrazolium bromide (MTT) and neutral red uptake (NRU) assays. MCF-7 cells were grown following the detailed method described in the supplementary information. The cell culture media (Dulbecco′s Modified Eagle′s Medium; DMEM) was supplemented with 7.81–250 µg/mL PB-extract capped Pd-NPs and sonicated (15 min at 40 W). MCF-7 cells (1 × 10^4^/mL) were then treated with NPs for 24 h in DMEM medium in microtiter wells of 96-well polystyrene plate. Experiment with bare surface Pd-NPs at 15.62, 31.25, 62.5 μg/mL was also run in parallel and the results were compared with PB-extract capped Pd-NPs. After incubation, DMEM was gently discarded and cells were rinsed with PBS (1X). MTT at 5 mg/mL rate was added to each well and incubated further for 4 h at 37 °C. MTT retained by cells was solubilized by 0.2 mL dimethyl sulfoxide (DMSO) absorbance at λ_max_ = 550 nm was recorded. Similarly, the NRU assay was performed. After the treatment of MCF-7 cells with Pd-NPs for 24 h, DMEM was supplied afresh carrying 50 μg/mL of neutral red. Further incubation of three hours was given. Microtiter wells containing this mixture were rinsed with a combination of HCHO and CaCl_2_ (mixed at a ratio of 0.5:1%). Thereafter, a mix of C_2_H_5_OH (50%) and CH_3_COOH (1%) was added to wells and incubated at 37 °C for 20 min. Absorbance at λ_max_ = 540 nm was recorded. Percent cell viability was plotted over untreated control with an increasing dose rate of PB-extract capped Pd-NPs.

To assess the apoptosis induced by Pd-NPs, MCF-7 cells were treated with 62.5 and 125 μg/mL concentration of Pd-NPs. Total RNA was extracted from cells and purified by a commercially available RNA purification kit procured from Roche, Mannheim, Germany as per manufacturer’s instructions. Extracted RNA was visualized following agarose gel electrophoresis (1%) and quantification of RNA was done by a NanoDrop spectrophotometer. The cDNA from RNAs was synthesized by using the Fermentas cDNA synthesis kit (Burlington, ON, Canada). cDNA synthesis was performed as per the protocol provided by the manufacturer. Primer sequences for housekeeping GAPDH gene (for normalization of gene expression) and four apoptotic genes namely *bax, p53*, *caspase-9,* and *caspase-3* are provided in the supplementary information (Table S1). PCR amplification of genes was performed in 35 cycles following the program: first cycle 95 °C for ten minutes; 35 cycles at 95 °C for 15 s, 60 °C for 30 s, and 72 °C for 30 s. DIW was used as a template for negative control. Gene expression data were analyzed by ^2-△△^Ct method fold changes in gene expression were compared with control.

### Data analysis

Each experiment was performed three times with triplicate samples for each test concentration. Data shows mean values and error bars represent standard deviation (S.D.). Significant differences between the values were calculated by student’s t-test at 95% confidence limit using Sigma Plot 14.0. All methods were carried out in accordance with relevant guidelines and regulations. Clinical cultures of bacteria were isolated from pus/wound samples of the registered patients and informed consent was obtained. Experimental protocols were approved by institutional committee of the University as and when required. No consent from ethical committee was required for sample collection for the isolation of bacteria.

### Ethics statement

All methods were carried out in accordance with relevant guidelines and regulations. Experimental protocols were approved by institutional committee of the Princess Nourah Bint Abdulrahman University, Registration No. H-01-R-059.

## Results and discussion

Many physiological or structural changes occur alone or simultaneously in bacteria when it is encountered by an antibiotic. The following are major antibiotic resistance mechanisms reported in clinical bacteria: (i) occurrence of bacterial mutations, (ii) horizontal gene transfer, (iii) destruction/modification of antibiotic molecules, (iv) decrease in cell membrane permeability to inhibit antibiotic penetration, (v) higher expression of efflux pumps in the membrane, and (vi) alteration of antibiotic target sites^34^. Moreover, bacterial species can form biofilms that is highly resistant towards antibiotics than planktonic cells, sometimes > 1000 folds^35^. On the other hand, the development of cancers and inefficient cure by available anti-cancer drugs further complicates their clinical treatments. Based on the drug sensitivity/resistance profile, cancers could be also be categorized as MDR cancer^36^. Therefore, environmentally sustainable, non-toxic, and cost-effective nano-therapeutics are in trend to treat such resistance in clinical bacteria and cancer cells.

**Figure 1:**
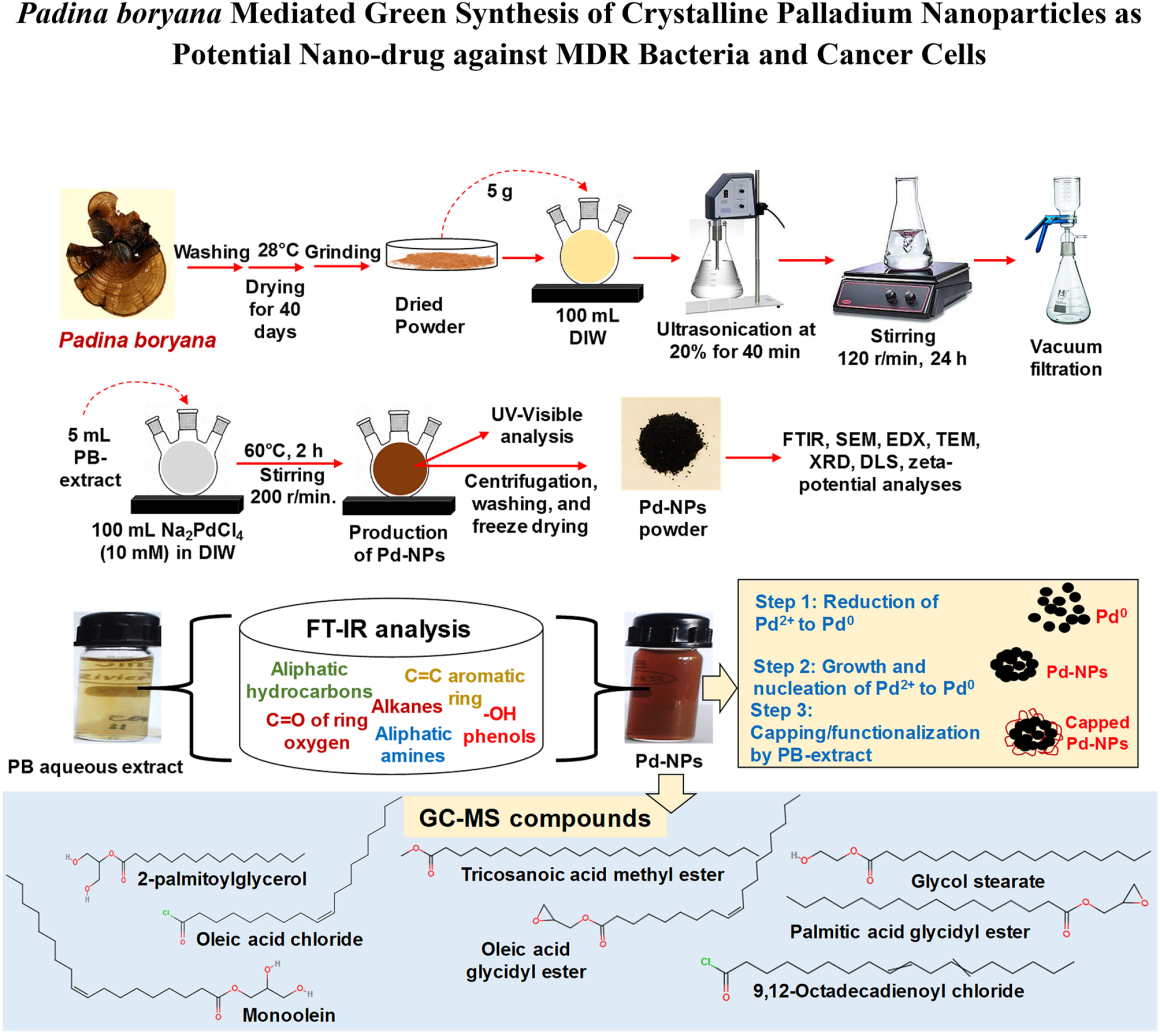
Scheme for *P. boryana* extract preparation, Pd-NPs fabrication, their characterization, and determination of capping by various techniques.

So far, few studies report the utilization of brown seaweed (marine algae) for Pd-NPs production^16,22^ even though the marine algal population is chemically rich possesses a wide range of compounds with promising anti-oxidant, anti-cancer, anti-inflammatory, and anti-microbial activities. The rich biomolecular composition of PB-extract and previous evidences on Pd-NPs biological activity prompted us to investigate the role of PB-extract in capping of Pd-NPs and interactions with six clinical bacteria and human breast cancer (MCF-7) cell line. The capping of NPs (i.e. adsorption of molecules on the surface of NPs) decides its final morphology and thus prevents the overgrowth of NPs. The method for synthesis and capping of Pd-NPs by PB-extract is detailed in Fig. 1 depicting (i) bio-reduction of Pd^2+^ ions to Pd^0^ seeds and (ii) capping/stabilization of Pd-NPs growth by molecules of PB-extract via surface adsorption. Production of Pd-NPs started following the reduction of Pd^2+^ to Pd^0^ from electrons liberated mainly from the reducing sugars and polyphenols containing biomolecules of *P. boryana* extract. As a result, the color of solution changed drastically from pale yellow to dark brown. This could be well corroborated with earlier observations^37^, where leaf biomolecules of *Solanum trilobatum* while interacting with Pd^2+^ ions gave dark precipitation from the reaction mixture which could be due to surface plasmon resonance (SPR) owing to the collective oscillation of electrons. The biomolecules of PB-extract when donating electrons for Pd^2+^ ions reduction are oxidized and are expected to form intermediate Pd-organic complexes. Ions are then converted to Pd^0^ by free electrons generated in the medium^38^. Frequent collisions among Pd^0^ atoms lead to the production and growth of Pd-NPs that are meanwhile capped by other organics of PB-extract giving specific size and shape to growing Pd-NPs seeds.

### UV–vis spectroscopic analysis

**Figure 2:**
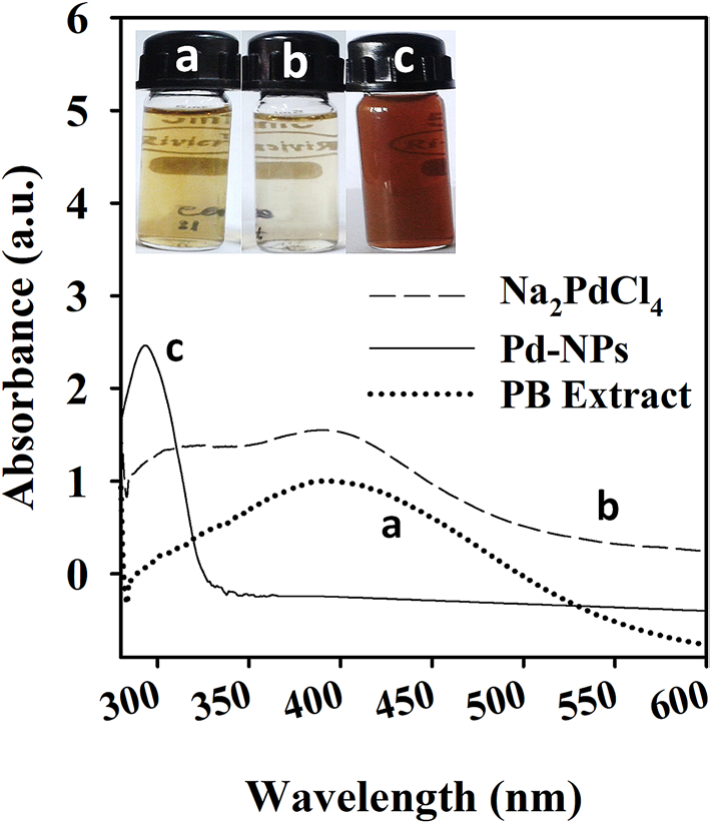
UV–Visible spectroscopic analysis of *P. boryana* aqueous extract (**A**), Na_2_PdCl_4_ (**B**), and Pd-NPs. Glass vials in inset represent the color change of PB-extract and Na_2_PdCl_4_ mixture after Pd-NPs synthesis.

NPs have optical features which give a preliminary idea about their shape, size, and SPR^39^. The comparative UV–vis spectra (Fig. 2) showed a broad absorption of PB-extract with a peak overlapping the UV and visible region. The precursor salt (Na_2_PdCl_4_) showed two characteristics peaks at 311 nm and 402 nm, while Pd-NPs exhibited a sharp peak at 293 nm. The absorption near to 293 nm by Pd-NPs has also been observed in other studies such as 268 nm^22^. In Na_2_PdCl_4_, two signals can be assigned to the transition of ligand to metal charge transfer between Pd^2+^ and Cl^-^. Absence of these two signals in the Pd-NPs spectrum advocates reduction of Pd^2+^ ions to NPs^40^ as also visually observed (Fig. 2 inset).

### Surface morphology and elemental composition of PB-capped Pd-NPs

**Figure 3:**
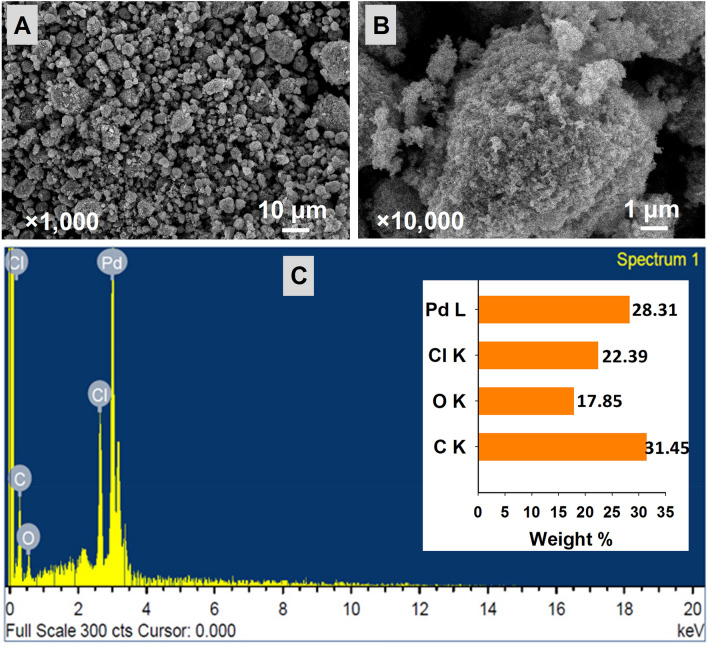
Determination of surface morphology of PB extract-capped Pd-NPs by SEM at × 1000 (**A**) and × 10,000 (**B**) magnification. Panelc C shows EDX analysis and elemental composition (inset of panel C) of synthesized Pd-NPs.

The morphological analysis of dehydrated powder of PB-capped Pd-NPs through SEM is shown in Fig. 3A,B at two different magnifications. Aggregates of variable sizes were recorded, however, the shape was pleomorphic. The elemental composition of Pd-NPs revealed the presence of Pd with carbon, oxygen, and chlorine (Fig. 3C). The Pd-NPs after synthesis were washed many times to remove the ions and unbound PB-extract, therefore, C, O, and Cl could appear in EDX spectra from the algal extract. The percentage of Pd was high in the EDX spectrum as 28.31% (Fig. 3C inset) and the peak clarity of EDX confirmed the purity of synthesized Pd-NPs. The peak at 3.1 keV in the EDX spectrum represents Pd as reported earlier^41^.

### Average Diameter, crystalline size, and structure of NPs

**Figure 4:**
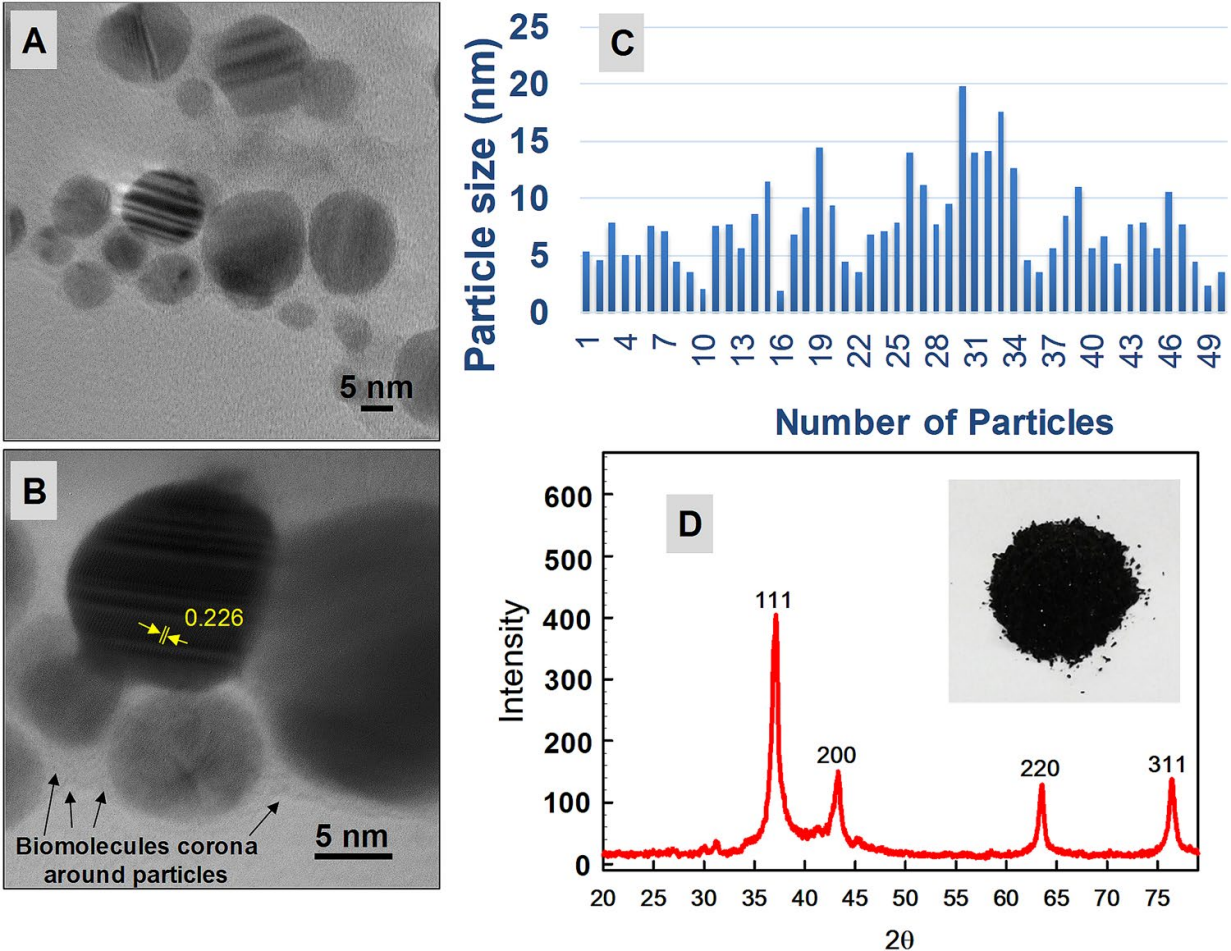
TEM micrographs of PB extract-capped Pd-NPs at direct magnification of 300000X (panel A) and  × 800,000 (panel B). Panel C shows particle size distribution while panel D represent XRD spectra with inset image of Pd-NPs powder.

The TEM micrographs showed spherical shape Pd-NPs (Fig. 4A,B) with a narrow range of particle size distribution from 5 to 20 nm (Fig. 4C). The average particle diameter calculated from the TEM size distribution was 8.7 nm. Pd-NPs were found well dispersed and agglomeration was absent. There was least direct particle-to-particle adherence and nil fusion of Pd-NPs during TEM analysis that could be due to the corona formation by biomolecules of PB-extract during capping. In Fig. 4B, the lattice *d*-spacing of 0.226 nm is shown while the distance between planes was measured. This *d*-spacing confirmed the crystallinity of PB-capped Pd-NPs. The XRD pattern of Pd-NPs (Fig. 4D) revealed one major (111) and three minor signals (200), (220), and (311) which were sharp and intense. The crystalline plane of (111) was well-matched with Pd (*d*-spacing of 0.23 nm) and revealed an FCC structure. The XRD derived average particle size was calculated as 11.16 nm by using Debye–Scherrer's equation which is good agreement with TEM size. Peaks could corroborate with the standard JCPDS file of crystalline Pd (file no. 05-0681)^42^. Thus XRD and TEM data confirmed the purity and crystalline nature which is consistent with other plant and green algae mediated fabrication studies of Pd-NPs^16,43^, however, the antibiofilm and anti-cancer activities were lacking.

### Surface area, hydrodynamic size, and zeta-potential

**Figure 5:**
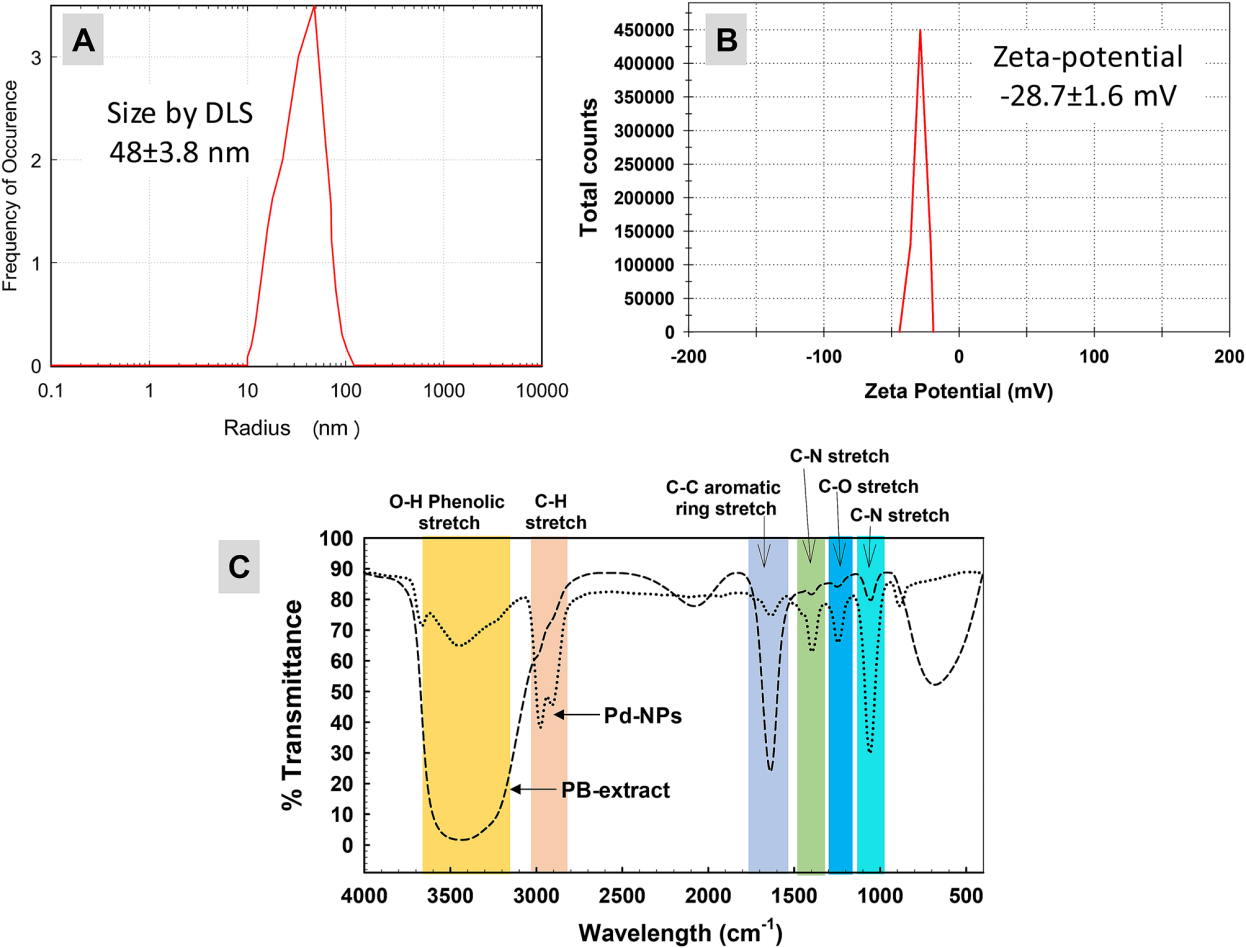
Hydrodynamic size of PB extract-capped Pd-NPs recorded by DLS (panel A) and zeta-potential (panel B). Panel C shows comparative analysis of FT-IR spectra of PB extract and Pd-NPs.

The surface area recorded for Pd-NPs was 16.1 m^2^/g. DLS analysis revealed the average hydrodynamic size of Pd-NPs as 48 ± 3.8 nm while zeta-potential was found as − 28.7 ± 1.6 mV (Fig. 5A,B). The increase in size compared to the primary size measured by TEM and XRD shows some sort of particle aggregation in the aqueous solution which depends on the frequency of NPs collisions as inter-particular interactions increases. Due to these collisions, the average path length covered by NPs decreases thereby increasing the hydrodynamic size^44^. A similar kind of variation has been seen in another study where size of Pd-NPs measured by DLS increased up to 24.20 nm as compared to size (4 nm) measured by TEM^38^ which was suggested due to the presence of biomolecules from *Delonix regia*. The stability of Pd-NPs was assessed by determining the zeta-potential that gives information about surface electrostatic potential and movement of NPs in the suspension. The zeta-potential of − 28.7 ± 1.6 mV denotes sufficient stability of Pd-NPs for effective biological applications. This could probably be due to the efficient capping of Pd-NPs by biomolecules of algal extract producing repulsion among Pd-NPs in solution^45^.

### Surface functional groups/biomolecules analyzed by FT-IR and GC–MS

The IR signals detected for extract and NPs (Fig. 5C) are assigned to various functional groups in Table 1. All the major signals detected for extract were also found in Pd-NPs except one which was for C-H stretch at 2086 cm^−1^. The FT-IR spectrum of Pd-NPs suggests the possible role of –OH functional groups having compounds such as polyols including terpenoids, tannins, saponins, etc.^46^ forming complex with Pd-NPs as revealed by narrowing of the peak. The shift in transmittance of FT-IR signals of Pd-NPs as compared to extract could be due to possible interactions of functional groups with metal ions (during reduction) and atoms or smaller NPs (during capping) as observed in the current study ^47^. The signals at 2986, 1643, and 1062 cm^−1^ of extract were shifted to 2974, 1637, and 1059 cm^−1^ in Pd-NPs after bio-reduction suggesting the involvement of aliphatic hydrocarbons, aromatic rings, aliphatic amines^48,49^.

**Table 1:** FT-IR investigation of functional group signals and bond assignment.

Functional group vibration	IR signal in PB-extract	IR signal in Pd-NPs	References
‒OH phenolic stretch	3507–3335	3468–3417	Bagad and Khan (2015)
C‒H stretch of aliphatic hydrocarbons	2986	2974	Ali et al. (2015)
C–H stretch	2086	-	Paluszkiewicz and Kwiatek (2001)
C=C aromatic ring stretch	1643	1637	Wang et al. (2014)
C–N stretch and C-H bending of alkanes	1402	1401	Balaji et al. (2017)
C–O stretch of ring-oxygen	1247	1247	Bagad and Khan (2015)
C–N stretch of aliphatic amines	1062	1059	Wang et al. (2014)

The role of PB-extract organic molecules in the formation and encapsulation of Pd-NPs was investigated by GC–MS analysis. Various biomolecules were detected in GC–MS and compared with those present in PB-extract capped Pd-NPs. GC–MS analysis of the extract revealed the presence of 31 major and minor peaks (Table S2) which were identified as long-chain hydrocarbon acids, esters, and acid chlorides. The major compounds of extract were: 2-palmitoylglycerol (peak area 25.14%), tricosanoic acid, 2-methoxy-, methyl ester (peak area 19.46%), oleic acid glycidyl ester (peak area 6.78 + 4.91 = 11.78%), cinnamyl linoleate (peak area 4.87%), 9,12-Octadecadienoyl chloride (peak area 3.24%), oleic acid chloride (peak area 2.89%), methyl oleate (peak area 2.78%) and monoolein (1.49%). The variation in the structure of long-chain hydrocarbons is evident from linear molecules^50^ to cyclic hydrocarbons^51^ with various functional moieties such as –OH, > C=O, C–O–C, and –COOH as detected in the present study. Moreover, the single compound could appear as two or more individual peaks. This difference is due to the presence of stereoisomers, identical mass, and functional moieties. Under similar experimental conditions, the GC–MS analysis of Pd-NPs reflected 23 compounds similar to those in the extract (Table 2). The major among them were tricosanoic acid, 2-methoxy-, methyl ester (peak area 31.89%), 2-palmitoylglycerol (peak area 18.41%), oleic acid chloride (peak area 14.52%), oleic acid glycidyl ester (peak area 6.95%), glycol stearate (peak area 4.14%), monoolein (peak area 4%), 9,12-octadecadienoyl chloride (peak area 3.42%), and oleic acid, 3-hydroxypropyl ester (peak area 2.76%). These compounds were involved in the surface capping and stabilization of Pd-NPs. Similar to our study, long-chain aldehydes of various fatty acids such as palmitic, oleic, linoleic, and linolenic acids have been detected in red, green, and brown marine algae^52^. Likewise, an array of long-chain hydrocarbon fatty acids including oleic and palmitic acids were reported as abundant molecules in some species of Chlorophyta and Rhodophyta^53^. In another study, palmitic acid was also reported in brown algae by GC–MS analysis^54^. These type of compounds such as palmitic acid, stearic acid, linolenic acid, tetracosane, tetradecanoic acid, sitosterol etc. have also been detected in plants such as *Triticum aestivum*^55^, *Catharanthus roseus*^56^ and *Moringa oleifera*^56^ by GC–MS.

**Table 2:** GC–MS analysis of PB extract mediated Pd-NPs.

Peak	Peak area (%)	Retention time	Compound	Molecular formula	Molecular weight
1	0.59	12.235	12-Docosenol, TMS	C_25_H_52_OSi	396
2	0.78	14.985	Isobutyl Phthalate	C_16_H_22_O_4_	278
3	1.14	17.186	2,4-di-tert-butylphenol	C_14_H_22_O	206
4	0.76	18.562	Ethylene undecane dicarboxylate	C_15_H_26_O_4_	270
5	0.55	21.154	Methyl oleate	C_19_H_36_O_2_	296
6	18.41	21.569	2-palmitoylglycerol	C_19_H_38_O_4_	330
7	0.64	22.056	Palmitic acid, trimethylsilyl ester	C_19_H_40_O_2_Si	328
8	0.49	22.478	Hexadecanoic acid, dimethyl(isopropyl)silyl ester	C_21_H_44_O_2_Si	356
9	1.14	22.786	Monoolein	C_21_H_40_O_4_	356
10	0.89	22.569	Palmitic Acid Glycidyl Ester	C_19_H_36_O_3_	312
11	0.72	22.621	2,5-Di (Trifluoromethyl) Benzoic acid, Dodecyl Ester	C_21_H_28_F_6_O_2_	426
12	14.52	22.814	Oleic acid chloride	C_18_H_33_ClO	300
13	31.89	22.874	Tricosanoic acid, 2-methoxy-, methyl ester	C_25_H_50_O_3_	398
14	6.95	23.012	Oleic acid glycidyl ester	C_21_H_38_O_3_	338
15	4.14	23.478	Glycol stearate	C_20_H_40_O_3_	328
16	1.31	23.511	Palmitic acid glycidyl ester	C_19_H_36_O_3_	312
17	1.14	23.596	Cannabidiol	C_21_H_30_O_2_	314
18	2.18	24.104	Bis(2-ethylhexyl) phthalate	C_24_H_38_O_4_	390
19	3.42	24.314	9,12-Octadecadienoyl chloride	C_18_H_31_ClO	298
20	2.86	24.524	Monoolein	C_21_H_40_O_4_	356
21	0.93	24.789	Palmitic Acid Glycidyl Ester	C_19_H_36_O_3_	312
22	2.76	25.542	Oleic acid, 3-hydroxypropyl ester	C_21_H_40_O_3_	340
23	1.79	26.142	Fumaric acid, decyl 2-heptyl ester	C_21_H_38_O_4_	354
	**100**				

### Identification of bacterial pathogens and drug resistance

**Figure 6:**
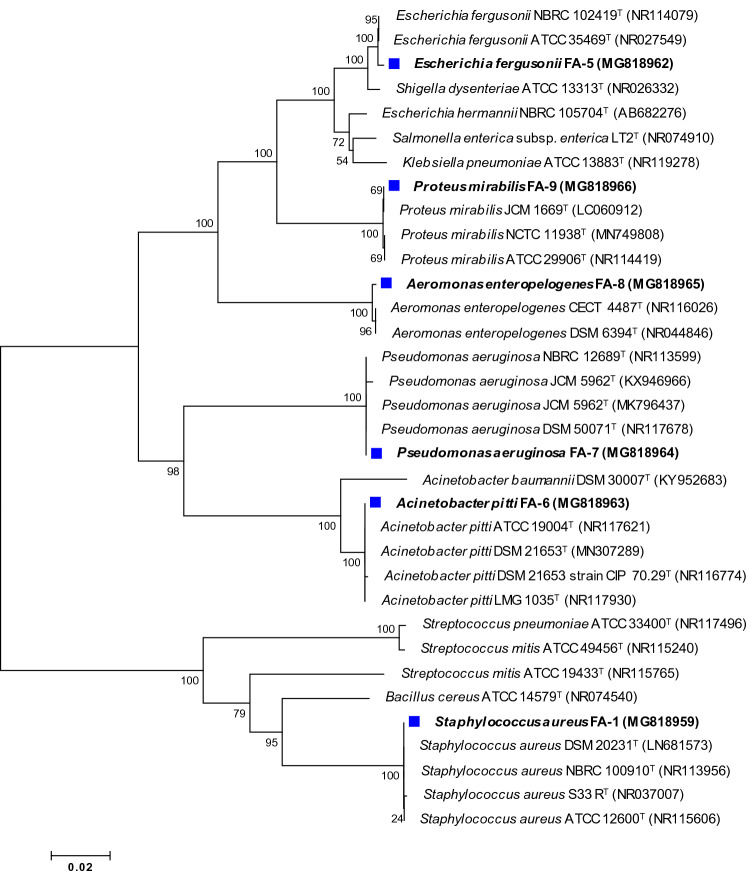
Unrooted neighbour-joined phylogenetic tree of isolated bacteria. The tree was constructed based on 16S rRNA partial gene sequence of bacterial isolates (marked with blue square) and closely related type species derived using NCBI BLAST search tool. Sequences were aligned using Clustal W sequence alignment tool in MEGA 7.0 software. The GenBank accession numbers of isolates and closely related species are placed in parenthesis. Bootstrap percentage values as obtained from 1000 replications of the data set are given at tree’s nodes. The scale bar corresponds to the mean number of nucleotide substitutions per site.

*S. aureus* showed a Gram-positive reaction whereas, *E. fergusonii*, *A. pitti*, *P. aeruginosa*, *A. enteropelogenes*, and *P. mirabilis* were found Gram-negative. Molecular identification by partial sequencing of 16S rDNA and phylogenetic analysis based on comparison with type strains (Fig. 6) revealed that strain FA-1, FA-5, FA-6, FA-7, FA-8, and FA-9 were *S. aureus, E. fergusonii, A. pitti, P. aeruginosa*, *A. enteropelogenes*, and *P. mirabilis*, respectively. The percent similarity of test strains with standard bacteria and obtained accession numbers are presented in Table S3. Antibiotic profiling of test strains showed 69.5, 47.8, 78.2, 86.9, 60.8, and 56.5% resistance by *S. aureus*, *E. fergusonii*, *A. pitti*, *P. aeruginosa*, *A. enteropelogenes*, and *P. mirabilis* towards different classes of β-lactam and non-β-lactam antibiotics (Table 3). These strains isolated from various sources have also been found drug-resistant to different sets of antibiotics in other studies^57–61^.

**Table 3:** Antibiotic resistance profile of bacterial isolates.

Antibiotics			Disc potency (µg/disc)	Zone of inhibition (mm)					
				*S. aureus* FA-1	*E. fergusonii* FA-5	*A. pittii* FA-6	*P. aeruginosa* FA-7	*A. enteropelogenes* FA-8	*P. mirabilis* FA-9
	Antimicrobial class	Antibiotic							
β-lactam antibiotics	Cephems	Cefa-sulbactum	75/10	19^S^	20^S^	13^R^	15^R^	19^S^	12^R^
		Cefoperazone	75	12^R^	15^R^	17^I^	14^R^	13^R^	14^R^
		Cefepime tazobactam	30/10	19^S^	18^S^	14^R^	18^S^	14^R^	11^R^
		Cefotaxime	30	13^R^	17^R^	13^R^	13^R^	17^S^	13^R^
		Ceftazidime	30	12^R^	16^R^	11^R^	15^I^	13^R^	11^R^
		Cefepime	30	12^R^	17^R^	16^I^	13^R^	27^S^	18^I^
	Carbapenem	Imipenem	10	16^I^	21^S^	13^R^	13^R^	22^S^	20^S^
	Monobactam	Aztreonam	30	18^I^	16^R^	11^R^	12^R^	20^S^	17^I^
	Penicillin-like antibiotics	Amoxicillin	10	0^R^	15^I^	10^R^	11^R^	11^R^	10^R^
	Penicillin beta-lactam antibiotic	Oxacillin	5	14^R^	17^I^	12^R^	0^R^	0^R^	11^R^
Non β-lactam antibiotics	Sulfonamides	Co-trimoxazole	25	0^R^	15^I^	16^I^	12^R^	0^R^	10^R^
	Fluoroquinolones	Ciprofloxacin	5	0^R^	13^R^	12^R^	10^R^	10^R^	15^I^
		Norfloxacin	10	18^S^	14^R^	0^R^	0^R^	10^R^	19^S^
		Gatifloxacin	5	0^R^	12^R^	10^R^	0^R^	17^I^	10^R^
		Ofloxacin	5	0^R^	0^R^	0^R^	11^R^	12^R^	10^R^
	Aminoglycosides	Gentamicin	10	13^R^	15^S^	19^S^	17^S^	20^S^	18^S^
		Amikacin	30	14^R^	14^R^	12^R^	12^R^	10^R^	16^I^
		Tobramycin	10	14^R^	14^I^	15^I^	12^R^	0^R^	17^I^
	Macrolides	Erythromycin	15	0^R^	11^R^	0^R^	0^R^	15^I^	0^R^
	Ansamycin	Rifampicin	5	23^I^	18^I^	10^R^	0^R^	16^I^	0^R^
	Tetracycline	Tetracycline	30	17^I^	14^I^	11^R^	10^R^	10^R^	17^I^
	Quinolones	Nalidixic acid	30	13^R^	15^I^	10^R^	12^R^	0^R^	10^R^
	Phenicols	Chloramphenicol	30	0^R^	15^I^	0^R^	11^R^	0^R^	16^I^
	% Resistance			69.5	47.8	78.2	86.9	60.8	56.5

*S* sensitive, *I* intermediate, *R* resistant based on Clinical and Laboratory Standards Institute (CLSI) and the European Committee for Antimicrobial Susceptibility Testing (EUCAST) guidelines. Percent (%) resistance was calculated using following formula = total no. of antibiotics marked with R for each strain/total no. of test antibiotics × 100.

### Zone of inhibition and MIC of PB-extract capped Pd-NPs

Bacteria were not found sensitive to PB-extract, however, the zone of inhibition produced by gentamicin and Pd-NPs were variable among test strains (Table 4). The size of the inhibition zone created by Pd-NPs was higher than positive control for each bacterium. Recently, a similar kind of inhibition zone was produced by *Rosmarinus officinalis* mediated Pd-NPs on three clinical bacteria *Micrococcus luteus*, *S. aureus*, and *S. epidermidis* as compared to the positive control, ciprofloxacin^62^. MIC was variable i.e. 125 μg/mL for *S. aureus* and *P. mirabilis*, whereas, 62.5 μg/mL for other strains (Table 5). In a similar study, the MIC of Pd-NPs (prepared by *Sapium sebiferum* leaf extract) for *S. aureus*, *P. aeruginosa*, and *Bacillus subtilis* was measured as 45.4, 103.5, and 71.2 μg/mL^63^ which is in fair agreement with our results.

**Table 4:** Antibacterial activity of PB-capped Pd-NPs by well diffusion assay.

Bacterial isolates	Zone of inhibition (mm)		
	Negative control (PB extract)	Positive control (Gentamicin 10 µg/disc)	PB-capped Pd-NPs (100 µL/well from stock of 1 mg/mL)
*Staphylococcus aureus*	0 ± 0	13.3 ± 0.47	18.3 ± 1.24
*Escherichia fergusonii*	0 ± 0	15.3 ± 0.47	20.0 ± 0.81
*Acinetobacter pittii*	0 ± 0	19.0 ± 0.81	23.0 ± 0.8
*Pseudomonas aeruginosa*	0 ± 0	15.6 ± 0.94	21.3 ± 0.47
*Aeromonas enteropelogenes*	0 ± 0	20.6 ± 0.9	19.3 ± 0.5
*Proteus mirabilis*	0 ± 0	18.3 ± 0.47	23.0 ± 1.6

**Table 5:** Minimum inhibitory concentration (MIC) of PB-capped Pd-NPs.

Bacterial isolates	MIC (µg/mL)
*Staphylococcus aureus*	125
*Escherichia fergusonii*	62.5
*Acinetobacter pittii*	62.5
*Pseudomonas aeruginosa*	62.5
*Aeromonas enteropelogenes*	62.5
*Proteus mirabilis*	125

### Pd-NPs concentration-dependent growth of isolates and membrane destruction

**Figure 7:**
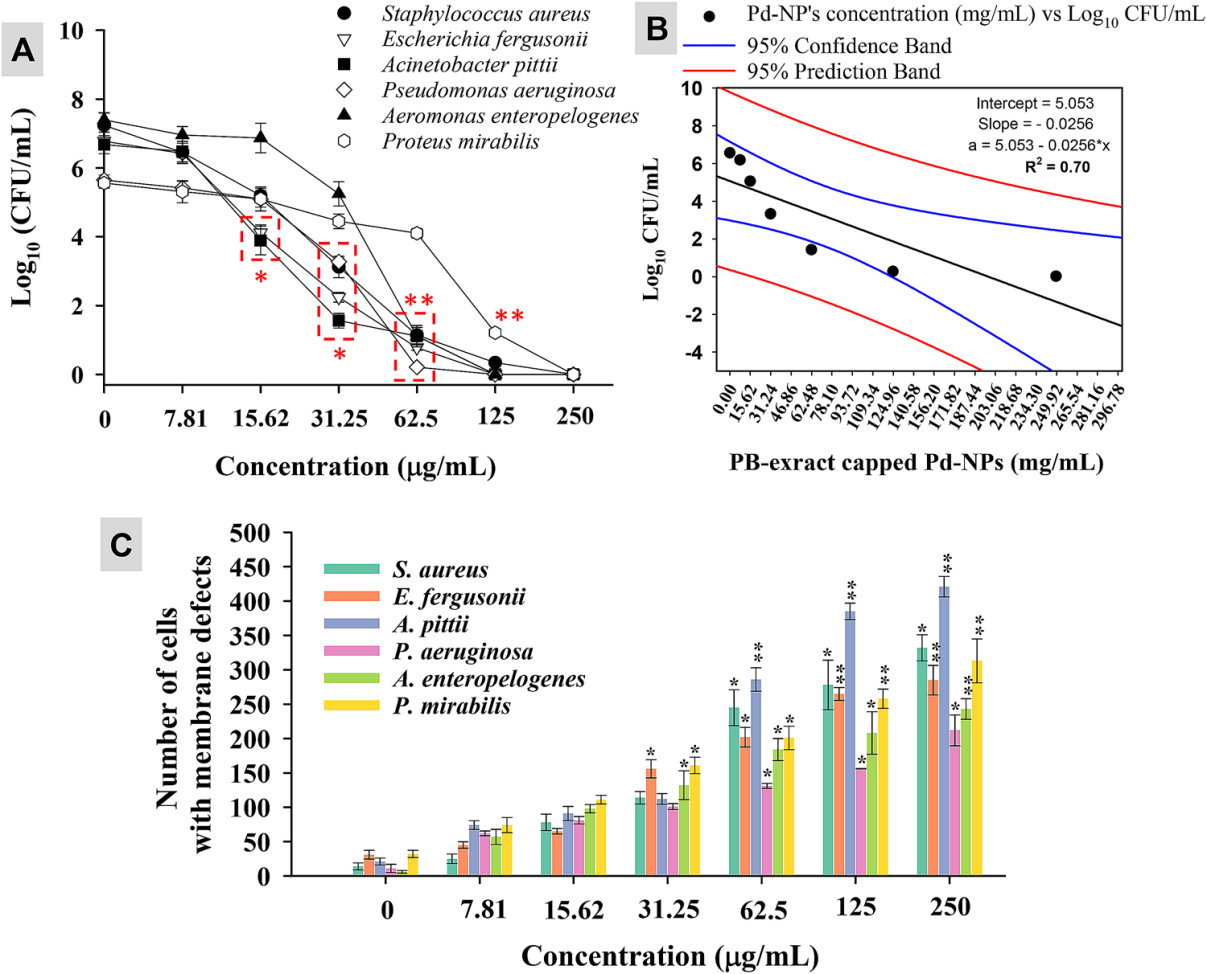
PB extract-capped Pd-NPs concentration (7.81–250 µg/mL) dependent CFU count of bacterial cultures (panel A), regression analysis of average CFU/mL of all strains versus PB extract-capped Pd-NPs concentration (panel B), and impact of PB extract-capped Pd-NPs on cell membrane permeability (panel C). Data represents mean values of three independent replicates and error bars represent standard deviation (S.D.). *P ≤ 0.05 and **P ≤ 0.01 based on student’s t-test.

When the exposure of Pd-NPs was increased from 7.81 μg/mL at a geometrical progression with a common ratio of 3 up to 250 μg/mL, the number of viable cells and thus CFU/mL at logarithmic scale was reduced (Fig. 7A). A ≤ 50% reduction in cell population was observed at 31.25 μg/mL for *E. fergusonii, S. aureus*, and *A. pitti*, 62.5 μg/mL for *A. enteropelogenes* and *P. aeruginosa,* and 125 μg/mL for *P. mirabilis*. Regression analysis between concentrations of Pd-NPs versus average Log_10_ CFU/mL resulted in an R^2^ value of 0.70 which shows a negative correlation (Fig. 7B). So far no study has reported the CFU-based bacterial inhibition by algal mediated Pd-NPs. Progressive damage to the bacterial cell membrane permeability was also noticed (Fig. 7C). PI clearly distinguishes between live and dead cell due to its ability to permeate only the membrane damaged cells followed by binding to DNA and fluorescence emission (λexc = 532 nm). Cells exposed to low concentration of Pd-NPs (7.81–15.62 μg/mL) showed less number of membrane altered cells. However, a significant (*P* ≤ 0.05 or 0.01) number of membrane compromised cells at a higher dose rate suggests substantial interaction of Pd-NPs with the bacterial cell membrane.

### Biofilm inhibition of isolates by PB-capped Pd-NPs

**Figure 8:**
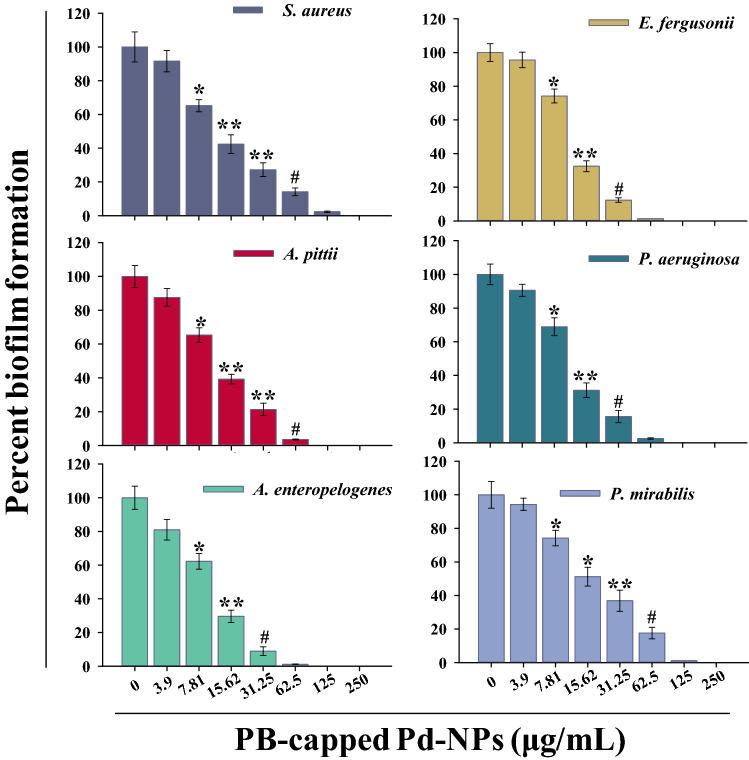
PB extract-capped Pd-NPs (3.9–250 µg/mL) dependent biofilm formation by bacterial cultures over respective untreated controls. Data represents mean values of three independent replicates and error bars represent standard deviation (S.D.). *P ≤ 0.05, **P ≤ 0.01, #*P* ≤ 0.001 based on student’s t-test.

Biofilm formation of test strains was first compared with biofilm positive strains and it was found that all the MDR strains could form the biofilm (Figure S1). PB-extract capped Pd-NPs significantly reduced the biofilm formation in a dose-related fashion as compared to control (Fig. 8). For the sake of comparison, at 31.25 μg/mL concentration, the order of biofilm formation was: *P. mirabilis* (36.9%) > *S. aureus* (27.2%) > *A. pitti* (21.36%), *P. aeruginosa* (15.63%) > *E. fergusonii* (12.36%) > *A. enteropelogenes* (8.9%). At 125 μg/mL, Pd-NPs completely abolished the biofilm formation irrespective of the test strain. Biofilms play a very critical role in successfully producing virulence factors that are controlled by a feed-forward control loop of quorum sensing signals (production of acyl-homoserine lactones) and thus a drug-resistant infection establishes^64^. To date, no antibiofilm study of marine algae-mediated Pd-NPs has been reported, however, a nanocomposite of Pd-graphene oxide showed a reduction in biofilm formation of *Bacillus subtilis*, *E. coli*, *P. aeruginosa*, *Klebsiella pneumoniae* by microdilution method^65^. Besides, extra polymeric substance (EPS), biofilms also contain lipids, proteins, and DNA that resist higher concentrations of antibiotics and compromise the host immune system.

**Figure 9:**
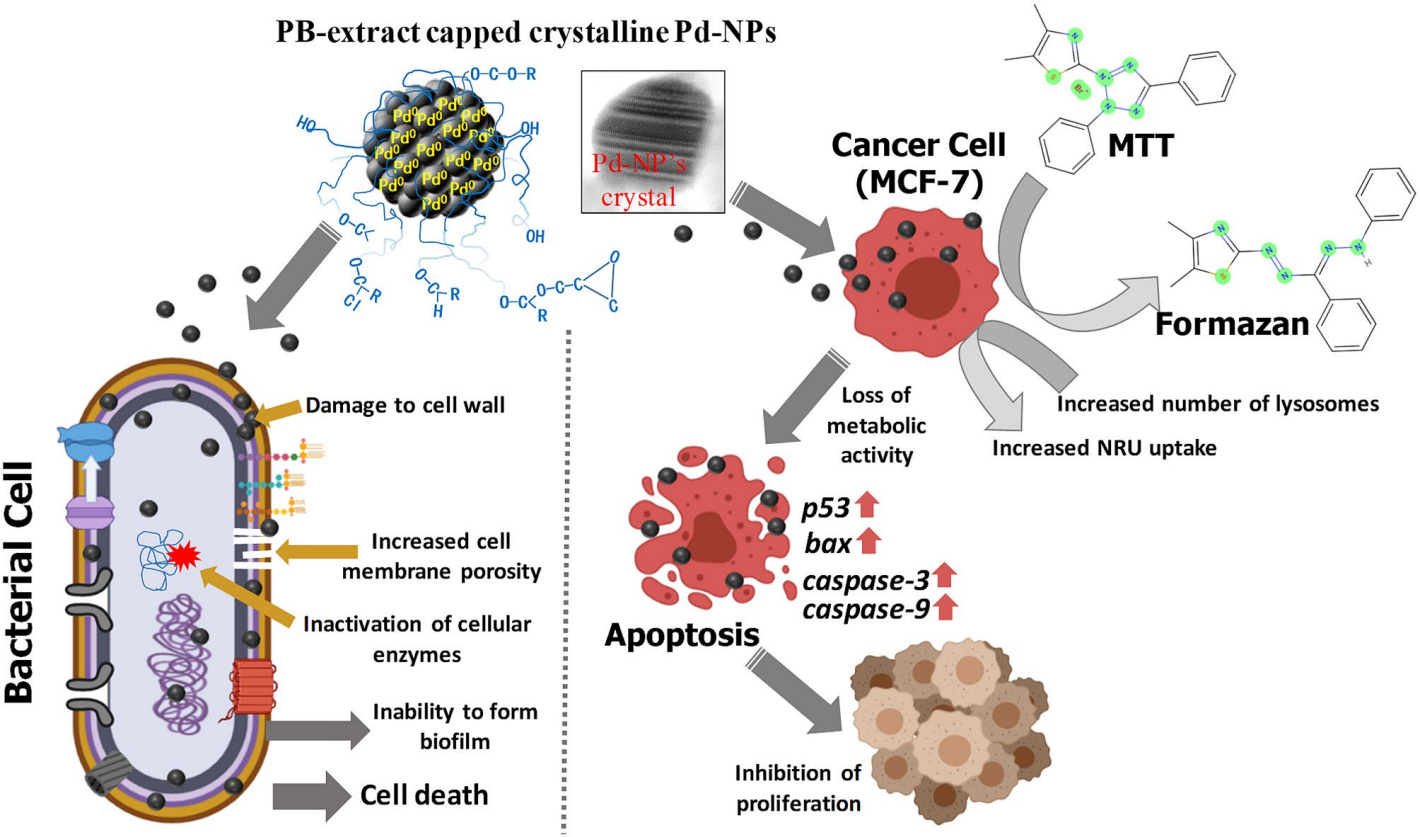
Schematic representation of PB-extract capped Pd-NPs mediated bacterial and cancer cell death.

The smaller size Pd-NPs synthesized in our study with a large surface area and biologically active capping material could serve as an alternative or supplement to antibiotics effectively inhibiting the growth of pathogens. While interacting with bacterial cells, PB-extract capped Pd-NPs can exert toxic impacts on bacterial growth and metabolism by direct reactions including the following: (i) attachment to peptidoglycan (PG; a polymer of sugars and amino acids around cell membrane) layer due to the linkage between free amino groups (-NH_2_) and hydroxyl (-OH), carbonyl (> C=O), epoxide or ester groups of biomolecules present in PB-extract. This binding can also facilitate the inside entry of Pd-NPs into periplasmic space, (ii) creation of new pores in the cell membrane by physical interaction with phospholipids and membrane lipid peroxidation and thus altering the membrane permeability, (iii) inactivation of cellular enzymes, and (iv) destruction of biofilm formation by negatively charged EPS mediated mobilization of positively charged Pd-NPs or Pd^2+^ ions (released from Pd-NPs) to biofilms as depicted in (Fig. 9). Indirectly, encounter and binding of –SH groups of proteins with Pd-NPs can trigger a modification of PO_4_^–^ efflux system leading to cell membrane exfoliation from cytoplasm, intracellular oxidative stress, dysfunction DNA replication system, and leakage of cell content.

#### In vitro cancer cell cytotoxicity and apoptosis

**Figure 10:**
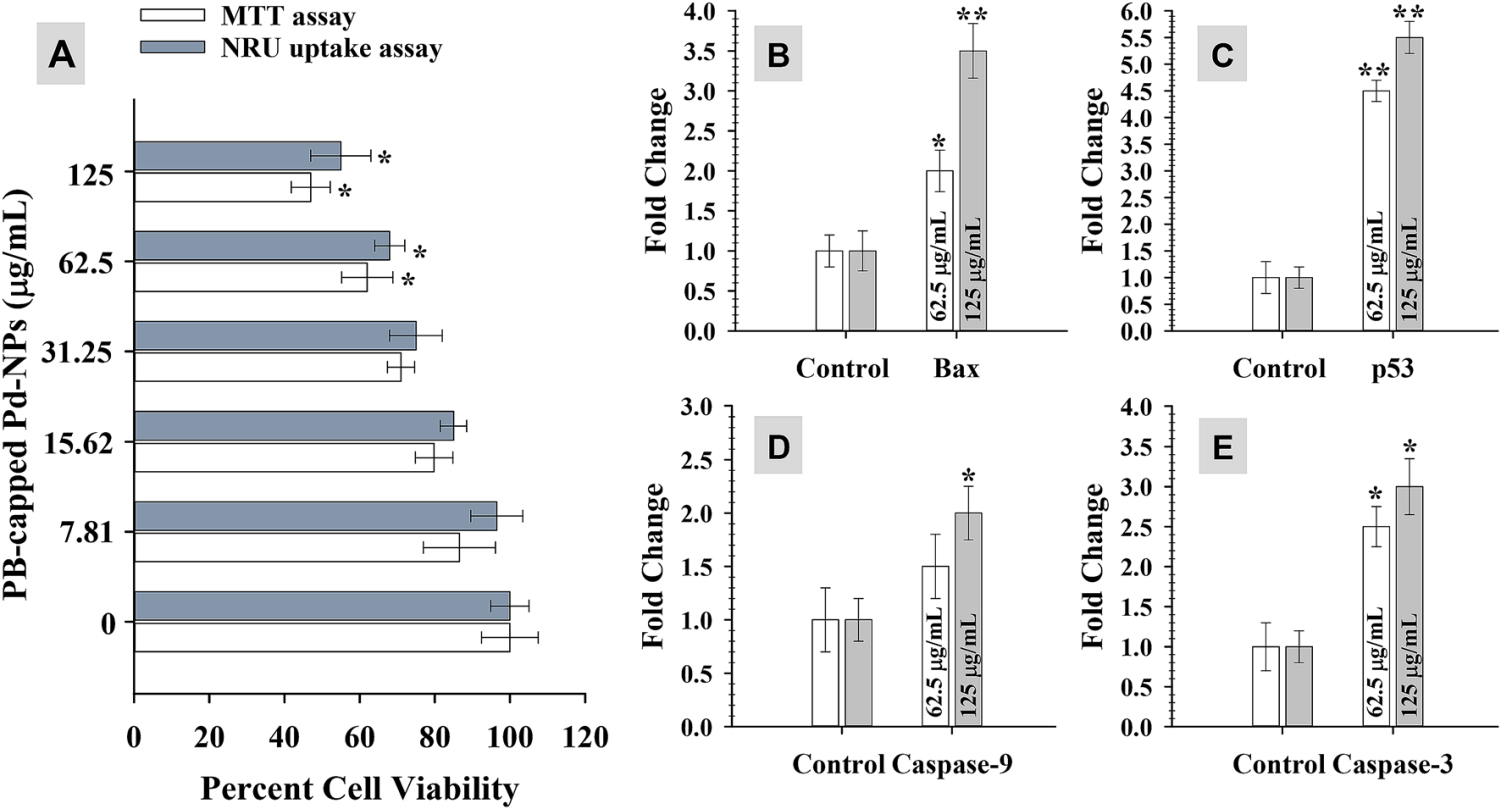
Anti-cancer activity of PB extract-capped Pd-NPs in a concentration (7.81–250 µg/mL) dependent manner as measured by MTT and NRU assays (panel A). Panels B-E shows fold changes in gene expression of four apoptotic genes (*bax*, *p53*, *caspase-9*, and *caspase-3*). Data represents mean values of three independent replicates and error bars represent standard deviation (S.D.). *P ≤ 0.05 and **P ≤ 0.01 as calculated by student’s t-test.

MTT assay quantifies the production of cellular oxidoreductase enzymes (NADPH mediated) following the reduction of MTT stain and thus reveals the metabolic activity of cells. These enzymes reduce the MTT to an insoluble form of formazan. Whereas, NRU uptake assay measures the influx of neutral red into lysosomes of metabolically active cells. The data exhibited a dose-dependent significant (*P* ≤ 0.05 and 0.01) reduction of MCF-7 cellular activity and cell viability was found decreased as compared to control cells (untreated) (Fig. 10A). At 62.5 and 125 μg/mL of Pd-NPs, the percent reduction in viable cells was found as 38% and 53% by MTT assay, and 32% and 45% by NRU assay. This data clearly shows lysosomal toxicity and the destruction of cellular metabolism by Pd-NPs. The cytotoxic activity of green Pd-NPs to human leukemia cancer cell lines has been suggested due to the physicochemical interaction of Pd-NPs with DNA, proteins, phosphate groups, cell cycle arrest, free radical formation, and leakage of lactate dehydrogenase^66^. Under NPs stress, cancer cells regulate gene expression to circumvent cellular disruption thereby restoring signaling and cell cycle. In current study, two concentrations (62.5 and 125 μg/mL) of Pd-NPs induced expression of apoptotic marker genes in folds in the following order: *p53* (4.5-folds) > *caspase-3* (2.5-folds) > *bax* (2-folds) > *caspase-9* (1.5-fold) at 62.5 μg Pd-NPs/mL and *p53* (5.5-folds) > *bax* (3.5-folds) > *caspase-3* (3-folds) > *caspase-9* (2-folds) at 125 μg Pd-NPs/mL (Fig. 10 B–E). The enhanced expression of *p53* mRNA transcripts suggests multiple targets of Pd-NPs in MCF-7 cells including generation of oxidative stress, dysfunction of mitochondria, aberration on cell cycle, and apoptosis. Similarly, *bax* is a well-known apoptosis inducer. Higher expression of two of the major caspases (*caspase-9* and *caspase-3*) emphasizes the fragmentation of nuclear material and suggests the role of mitochondria in *p53* apoptosis. Moreover, higher expression of *p53* enhances the transcription of *bax*, *caspase-9,* and *caspase-3* as pro-apoptotic genes^67^. Cancer cell death mediated by PB-extract capped Pd-NPs is schematically presented in Fig. 9.

**Figure 11:**
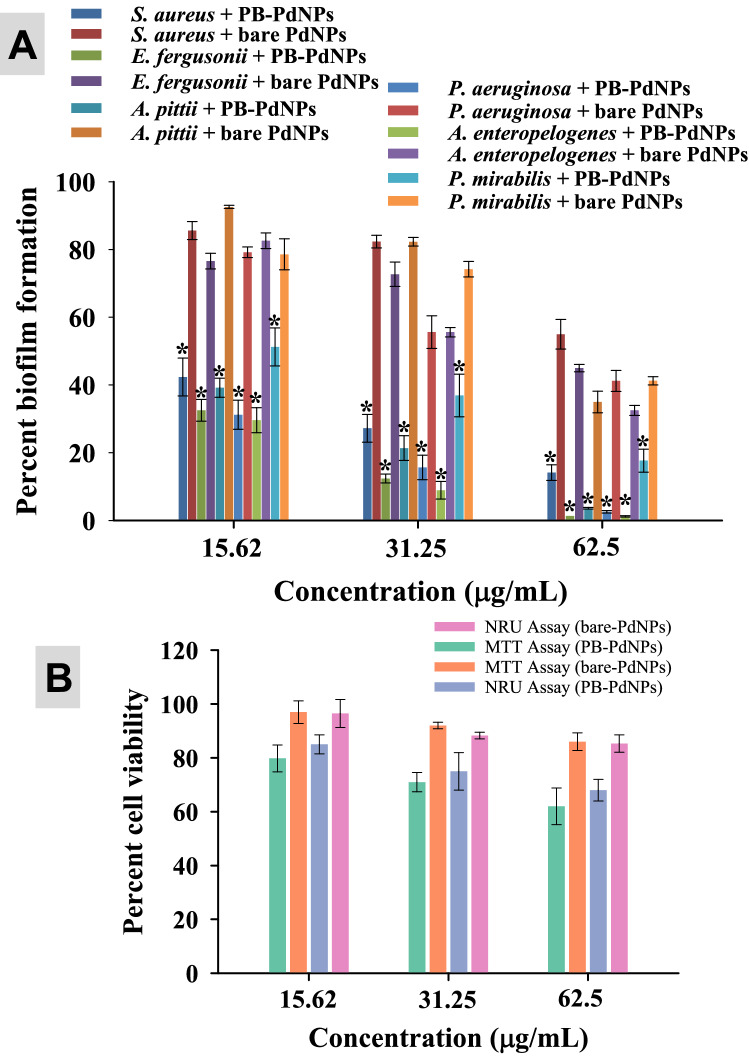
Comparison between the inhibiting potential of PB extract capped-PdNPs and bare surface Pd-NPs: Antibiofilm activity against six pathogenic strains (panel A) and viability of MCF-7 cells (panel B). Data represents mean values of three independent replicates and error bars represent standard deviation (S.D.). *P ≤ 0.05 as calculated by student’s t-test.

In a comparative analysis, PB-extract capped Pd-NPs were found more active against pathogenic biofilms and breast cancer cells as compared to bare-PdNPs Fig. 11. The enhanced in vitro clinical performance of PB-extract capped Pd-NPs is possibly due to the capping of *P. boryana* biomolecules. The apparent difference in antibiofilm and anticancer potential of two species of Pd-NPs probably arises as a result of bioactive corona of PB extract biomolecules and functional groups around Pd-NPs which might help in the enhanced uptake of PB-capped Pd-NPs by bacterial and cancer cells (primary factor) which in turn results in Pd-NPs toxicity (secondary factor). Similar kind of results have been reported in two other studies where green synthesized α-Fe_2_O_3_ and CuO NPs were compared with uncapped α-Fe_2_O_3_^68^ and CuO NPs^69^. Results showed substantial reduction of cell viability and biofilm formation by *E. coli*. *S. aureus*, and *P. aeruginosa*.

## Conclusion

The current study is perhaps the first study which explored the constituents of marine brown seaweed *P. boryana* by FT-IR and GC–MS analysis and proved their role in bio-reduction and bio-capping of Pd-NPs. The green synthesized Pd-NPs were fairly small in size, spherical and crystalline which were biologically effective in the range of 31.25–125 μg/mL against six MDR bacteria and human breast cancer MCF-7 cell line. The antibiofilm and anticancer efficiency of PB-extract capped Pd-NPs was higher than the uncapped or bare-PdNPs. A very few algae have been used for Pd-NPs fabrication but lack the detailed exploration of capping and assessment of biomedical potential. The developed synthesis method is cost-effective, green, and can be easily scaled up. Our green Pd-NPs are further warranted for in vivo research in an animal model to determine their safety to humans. However, the PB-extract capped Pd-NPs can be applied for the coating of medical appliances to control the resistant bacterial infections thus safeguarding the health of patients.

## Supplementary Information

Supplementary Information
