# Supplementary material for: Padina boryana mediated green synthesis of crystalline palladium nanoparticles as potential nanodrug against multidrug resistant bacteria and cancer cells
Source: Sci Rep. 2021 Mar 8;11:5444. doi: 10.1038/s41598-021-84794-6 (PMC7940407; doi:10.1038/s41598-021-84794-6)
Supplement: Supplementary file 1 — Supplementary Information [file 41598_2021_84794_MOESM1_ESM.docx]

***Padina boryana* Mediated Green Synthesis of Crystalline Palladium Nanoparticles as Potential Nano-drug against Multidrug-Resistant Bacteria and Cancer Cells**

Fuad Ameen^a,1^, Hana Sonbol^b, 1,^ Sami AlYahya^c^, Abobakr Almansob^a^ Suaad Alwakeel^b^

^a^Department of Botany & Microbiology, College of Science, King Saud University, Riyadh 11451, Saudi Arabia

^b^Department of Biology, College of Science, Princess Nourah Bint Abdulrahman University, Riyadh, Saudi Arabia.

^c^National center for Biotechnology, King Abdulaziz city for Science & Technology, Riyadh, Saudi Arabia

*Correspondence to: [fuadameen@ksu.edu.sa](mailto:fuadameen@ksu.edu.sa)

^1^Author contributed equally.

**Supplementary methods:**

**Phylogenetic analysis**

For phylogenetic analysis, BLASTn was performed with a limit set to only type materials. Thus, type bacterial strains showing the highest percent similarity (kinship) were downloaded in FASTA format and aligned using ClustalW pairwise and multiple alignment options in MEGA 6.0. Aligned sequences were used for phylogenetic tree construction using the Neighbour-joining method. Bootstrap percentage values as obtained from 1,000 replications of the data set were mentioned at nodes of phylogenetic tree.

**Culture of Human Breast Cancer Cell Line (MCF-7)**

The MCF-7 (Michigan Cancer Foundation-7) cells (ATCC; Manassas, USA) were cultured in Dulbecco′s Modified Eagle′s Medium (DMEM) supplemented with antibiotic solution (penicillin-streptomycin solution; 100 U/ml) and 10% Fetal Bovine Serum (FBS) was cultured in a CO_2_ incubator supplying 95% humidity, 5% CO_2_ at 37°C. Before GT-Fe_2_O_3_ nanoparticle exposure, the MCF-7 cells were sub-cultured by adding inoculum from the trypsinized (0.25 %) cells of 85 % confluent culture. The cells with 95 % viability were confirmed by a trypan blue dye exclusion assay.

**Supplementary Tables**

**Table S1:** Primer sequences used to synthesize cDNA.

| **Gene** | **Forward primer (5′ → 3′)** | **Reverse primer (5′ → 3′)** |
| --- | --- | --- |
| *GAPDH* | CCACTCCTCCACCTTTGAC | ACCCTGTTGCTGTAGCCA |
| *bax* | GCTGGACATTGGACTTCCTC | ACCACTGTGACCTGCTCCA |
| *p53* | CCCAGCCAAAGAAGAAACCA | TTCCAAGGCCTCATTCAGCT |
| *Caspase-9* | TACAGCTGTTCAGACTCTAGTA | AAATATGTCCTGGGGTAT |
| *Caspase-3* | TATGGTTTTGTGATGTTTGTCC | TAGATCCAGGGGCATTGTAG |

**Table S2:** GC-MS analysis of *P. boryana* extract.

| **Peak** | **Peak area (%)** | **Retention time** | **Compound** | **Molecular formula** | **Molecular weight** |
| --- | --- | --- | --- | --- | --- |
| 1 | 0.21 | 12.514 | 2,4-di-tert-butylphenol | C_14_H_22_O | 206 |
| 2 | 0.96 | 14.770 | 12-Docosenol, TMS | C_25_H_52_OSi | 396 |
| 3 | 0.46 | 18.147 | 3-methylcyclopentane-1,2-diol | C_6_H_12_O_2_ | 116 |
| 4 | 0.99 | 18.498 | Isobutyl Phthalate | C_16_H_22_O_4_ | 278 |
| 5 | 0.43 | 18.635 | Methyl Isoheptadecanoate | C_18_H_36_O_2_ | 284 |
| 6 | 0.52 | 18.674 | Methyl Octanoate | C_9_H_18_O_2_ | 158 |
| 7 | 0.65 | 19.147 | Palmitic acid, trimethylsilyl ester | C_19_H_40_O_2_Si | 328 |
| 8 | 1.96 | 19.268 | Ethylene undecane dicarboxylate | C_15_H_26_O_4_ | 270 |
| 9 | 1.18 | 19.521 | Hexadecanoic acid, dimethyl(isopropyl)silyl ester | C_21_H_44_O_2_Si | 356 |
| 10 | 1.43 | 20.186 | 9-Tetradecen-1-ol | C_14_H_28_O | 212 |
| 11 | 2.78 | 20.856 | Methyl oleate | C_19_H_36_O_2_ | 296 |
| 12 | 25.14 | 21.125 | 2-palmitoylglycerol | C_19_H_38_O_4_ | 330 |
| 13 | 1.68 | 21.745 | Palmitic Acid Glycidyl Ester | C_19_H_36_O_3_ | 312 |
| 14 | 1.12 | 22.145 | Monoolein | C_21_H_40_O_4_ | 356 |
| 15 | 1.78 | 22.550 | 2,5-Di (Trifluoromethyl) Benzoic acid, Dodecyl Ester | C_21_H_28_F_6_O_2_ | 426 |
| 16 | 0.84 | 22.601 | Fumaric acid, decyl 2-heptyl ester | C_21_H_38_O_4_ | 354 |
| 17 | 2.08 | 22.647 | Glycol stearate | C_20_H_40_O_3_ | 328 |
| 18 | 2.63 | 22.796 | 17-Octadecynoic acid, tertbutyldimerthylsilyl ester | C_24_H_46_O_2_Si | 394 |
| 19 | 6.78 | 22.921 | Oleic acid glycidyl ester | C_21_H_38_O_3_ | 338 |
| 20 | 19.46 | 22.956 | Tricosanoic acid, 2-methoxy-, methyl ester | C_25_H_50_O_3_ | 398 |
| 21 | 4.87 | 23.142 | Cinnamyl linoleate | C_27_H_40_O_2_ | 396 |
| 22 | 2.89 | 23.254 | Oleic acid chloride | C_18_H_33_ClO | 300 |
| 23 | 2.63 | 23.698 | Bis(2-ethylhexyl) phthalate | C_24_H_38_O_4_ | 390 |
| 24 | 1.64 | 23.714 | (Z)6,(Z)9-Pentadecadien-1-ol | C_15_H_28_O | 224 |
| 25 | 4.91 | 24.345 | Oleic acid glycidyl ester | C_21_H_38_O_3_ | 338 |
| 26 | 2.58 | 24.571 | Butoxy(cyclohexyl)dimethylsilane | C_12_H_26_OSi | 214 |
| 27 | 0.37 | 25.126 | Monoolein | C_21_H_40_O_4_ | 356 |
| 28 | 1.2 | 25.354 | Cannabidiol | C_21_H_30_O_2_ | 314 |
| 29 | 1.21 | 26.478 | Dinonyl ketone | C_19_H_38_O | 282 |
| 30 | 3.24 | 27.744 | 9,12-Octadecadienoyl chloride | C_18_H_31_ClO | 298 |
| 31 | 1.38 | 28.472 | Beta-sitosterol | C_29_H_50_O | 414 |
|  | **100** |  |  |  |  |

**Table S3:** Percent identity of partial gene sequence of 16S rRNA of isolated bacterial strains with that of type strains as revealed by BLAST search of NCBI database.

| **S. No.** | **Bacterial strain** | **GenBank accession number** | **Percent identity with type strains (%)** |
| --- | --- | --- | --- |
| 1 | *Staphylococcus aureus* FA-1 | MG818959 | - *Staphylococcus aureus* NBRC 100910 (99.93%) - *Staphylococcus aureus* ATCC 12600 (99.93%) - *Staphylococcus aureus* DSM 20231T (99.87%) - *Staphylococcus aureus* S33 R (99.73%) |
| 2 | *Escherichia fergusonii* FA-5 | MG818962 | - *Escherichia fergusonii* ATCC 35469 (99.45%) - *Escherichia fergusonii* NBRC 102419 (99.32%) |
| 3 | *Acinetobacter pittii* FA-6 | MG818963 | - *Acinetobacter pittii* DSM 21653 (100.00%) - *Acinetobacter pittii* LMG 1035 (99.93%) - *Acinetobacter pittii* ATCC 1904 (99.86%) |
| 4 | *Pseudomonas aeruginosa* FA-7 | MG818964 | - *Pseudomonas aeruginosa* JCM 5962 (100.00%) - *Pseudomonas aeruginosa* NBRC 12689 (100.00%) - *Pseudomonas aeruginosa* DSM 50071 (99.80%) - *Pseudomonas aeruginosa* JCM 5962T (99.72%) |
| 5 | *Aeromonas enteropelogenes* FA-8 | MG818965 | - *Aeromonas enteropelogenes* CECT 4487 (99.73%) - *Aeromonas enteropelogenes* DSM 6394 (98.92%) |
| 6 | *Proteus mirabilis* FA-9 | MG818966 | - *Proteus mirabilis* JCM 1669 (100.00%) - *Proteus mirabilis* NCTC 11938 (99.80%) - *Proteus mirabilis* ATCC 29906 (99.80%) |

**Supplementary Figures**

**Fig. S1** Comparative analysis of biofilm formed (in terms of absorbance) by standard (ATCC) bacterial cultures and isolated strains. Data represents mean values of three independent replicates and error bars represent standard deviation (S.D.).
